# Psychological Determinants of Men’s Adherence to Cascade Screening for *BRCA1/2*

**DOI:** 10.3390/curroncol29040203

**Published:** 2022-04-02

**Authors:** Giulia Ongaro, Serena Petrocchi, Mariarosaria Calvello, Bernardo Bonanni, Irene Feroce, Gabriella Pravettoni

**Affiliations:** 1 Applied Research Division for Cognitive and Psychological Science, IEO, European Institute of Oncology IRCCS, 20141 Milan, Italy; serena.petrocchi@gmail.com (S.P.); gabriella.pravettoni@ieo.it (G.P.); 2 Department of Oncology and Hemato-Oncology, University of Milan, 20141 Milan, Italy; 3 Division of Cancer Prevention and Genetics, IEO, European Institute of Oncology IRCCS, 20141 Milan, Italy; mariarosaria.calvello@ieo.it (M.C.); bernardo.bonanni@ieo.it (B.B.); irene.feroce@ieo.it (I.F.)

**Keywords:** *BRCA1/2* mutations, male prevention, decision-making, genetic testing adherence, cascade screening

## Abstract

*BRCA1/2* germline mutations predispose carriers to an increased risk of breast, ovarian, prostate, pancreatic, and skin cancer. Men and women are equally likely to pass on or inherit the pathogenic variant. However, there is evidence that male relatives are less involved in cascade screening than female ones. At the same time, little attention has been given to the research on psychological determinants of men’s adherence to cascade screening in *BRCA1/2*-positive families. Applying some principles of the Health Action Process Approach model, the present research tested a model of relationships on the adherence to *BRCA1/2* cascade testing guidelines. The sample comprised 115 men’s first-degree relatives of women with verified germline mutations (Mage = 41.93; SD = 17.27). A pre–post test design was applied. Significant associations emerged between the intention to uptake *BRCA1/2* genetic testing and age, parental status, breast cancer risk perception, self-referred outcome expectancies, perceived benefit, coping self-efficacy, and planning. Higher perceived benefit predicted increases in intention, and higher intention and coping self-efficacy predicted increases in planning. Intention was a positive total mediator of the relationship between benefit and planning. On a theoretical level, our findings partially supported the Health Action Process Approach as a valuable model based on which interventions could be developed in the context of cascade screening for *BRCA1/2* genetic testing. Those results supported the importance of integrated genetic counselling sessions with a strict collaboration between geneticists and psychologists together with interventions planned to increase men’s self-monitoring ability to support their self-efficacy.

## 1. Introduction

Hereditary breast–ovarian cancer syndrome (HBOC-OMIM 604370, 612555) is an autosomal dominant condition caused by germline mutations in *BRCA1* (BReast CAncer 1 gene) and *BRCA2* (BReast CAncer 2 gene). HBOC is characterized by an increased risk for breast (both male and female) and ovarian cancer. Moreover, *BRCA1/2* carriers are also at a greater risk of developing other cancers, including prostate, pancreatic, and skin cancer [1,2,3]. Lifetime breast cancer risk is up to 1.2% in male *BRCA1* carriers and up to 8.8% in *BRCA2* carriers, compared to 0.1% in the general population [4,5]; the lifetime prostate cancer risk is higher in *BRCA2* than *BRCA1* carriers and the general population, specifically up to 20–25% [6]. Since HBOC is an autosomal dominant condition, men and women are equally likely to pass on or inherit the mutation [7].

Gender is considered a critical explanatory variable in understanding health-seeking behaviors, in particular in hereditary cancers conditions [8,9,10,11,12] in which specific “concerns and idealized notions of masculinity” impact men’s responses to health issues [8] (p. 422). Although men are as involved in *BRCA1/2* germline mutations as women, the common practice is to associate those mutations with female gender at most. In this vein, *BRCA1/2* is directly connected to breast cancer and the notion of masculinity, according to which only women have breast tissue, may prevent men from looking for information and help. Moreover, although males have those germline mutations as much as females, research has dedicated more attention to women compared to men [13].

Several factors may contribute to this bias. First, there are documented differences in how genetic tests are administered to male and female populations in routine clinical practice. Women are tested to search for *BRCA1* or *BRCA2* germline mutations, especially when diagnosed with early-onset breast and/or ovarian cancer. Lately, the National Comprehensive Cancer Network guidelines [6] suggested to test for *BRCA* germline mutations in male patients diagnosed with pancreatic or prostatic cancer. Indeed, until recently, men have been mainly involved in cascade screening, which is the process of testing at-risk relatives of a patient with an identified germline mutation [14].

In addition to the procedural considerations highlighted above, several psychological factors may be associated with lower *BRCA1/2* screening rates in men [15,16]. Men in *BRCA1/2*-positive families were less involved in every level of the counseling, testing, and communication process [8,15,16], compared to the female counterpart. There was evidence that the communication between males and female relatives proband (the first identified *BRCA1/2* family member; [17]) about *BRCA1/2* implication was poor [18]. Graves and colleagues [12] suggested that men may encounter specific challenges in discussing their breast cancer risk with female proband particularly, because they were often excluded from family conversations about familial cancer risk and were usually not informed of test results received by their female relatives. Other studies reported self-health concerns and fear of cancer in seeking testing among men in HBOC families [19,20,21], showing high avoidant and distancing coping strategies to maintain an emotional balance [20,21,22]. Few preliminary qualitative evidence showed that the reasons why men and women uptake genetic testing are similar and specifically related to: (a) concerns for the offspring, (b) a desire to learn more about their health conditions and to know how to manage the risk, (c) the level of perception of one’s own risk, (d) the perception of obligation by one’s own family, and (e) reproductive decisions [19,23]. However, some studies focusing only on men’s experience showed that the male’s decision is frequently perceived as a family duty or an obligation towards their children [19,22,24], as if decision-making about *BRCA1/2* genetic testing was a family affair rather than an individual choice, requiring a less individualistic approach [23,25].

In the attempt to explain the engagement in healthy preventative behaviors, such as undergoing *BRCA1/2* genetic testing, the Health Action Process Approach model (HAPA, [26]) proposed that two processes are involved. The first one consists in forming an intention to adopt a precautionary measure or change risk behaviors (motivation phase), which is followed by the planning of the action and then by the action itself (volition phase). In the motivation phase, among others, risk perception and outcome expectancies act as predisposing factors that lead to the formation of the intention. Specifically, risk perception refers to the perceived health threat that may stimulate the beginning of an action, and outcome expectancies refer to positive or negative evaluations about the outcomes of an action. However, good intentions do not always translate into behaviors [27,28,29]; for this to happen, it is necessary that the intention be transformed into a detailed plan of the desired action, supported by perceived self-efficacy in coping with interfering obstacles [26]. So, intention is considered as a middle-level mediator between the motivation phase and the volition phase. 

There is support for the use of the HAPA model in explaining the initiation and maintenance of health preventive behaviors in several health contexts [30,31,32,33] including cancer-related screening behavior [34,35]. To the best of our knowledge, no studies have quantitatively and systematically tested which psychological determinants are involved in men’s decision-making processes for predictive *BRCA1/2* genetic testing in cascade screening context. Therefore, the present research aimed at redressing this gap in the literature testing a model of relationships on first-degree males with family members tested positive for those pathogenic variants. Applying some principles of the HAPAmodel, the present research tested a model of relationships on the adherence to *BRCA1/2* cascade testing guidelines. Specifically, it was hypothesized that:

- Higher risk perceptions and outcome expectancies would predict higher intention to undergo genetic test for *BRCA* germline mutations after three weeks, controlling for socio-demographics and coping self-efficacy (hypothesis 1a–HP1a). Moreover, higher intention and coping self-efficacy would predict higher planning after three weeks, controlling for socio-demographics and the other psychological variables (hypothesis 1b–HP1b).
- Intention would serve as a mediator in the relationships between risk perceptions or outcome expectancies, and planning (hypothesis 2–HP2).

Based on qualitative results by Rauscher et al. [25] and Hallowell and colleagues [23], the present research differentiated outcome expectancies in self- and family referral. Therefore, an explorative research question was formulated: are there differences between self-referred outcome expectancies and family-referred outcome expectancies in their association with the intention to undergo *BRCA1/2* genetic testing? (research question 1—RQ1). Finally, since *BRCA1/2* pathogenic variants predispose carriers to an increased probability of different types of cancer, the research investigated which specifical risk perception (i.e., breast, pancreas, prostate cancer risk perception) is most associated with the intention to undergo genetic testing and if there are differences between the risk perceptions in their association with the intention, considering socio-demographic variables (research question 2—RQ2).

## 2. Materials and Methods

### 2.1. Procedure

The current study was approved by the local Ethics Committee of the European Institute of Oncology (IEO) (approval number R1249/20-IEO 1314). Firstly, female patients who underwent genetic counselling and *BRCA1/2* testing at the Division of Cancer Prevention and Genetics of the European Institute of Oncology between 1998 and 2019 and were carrier of at least one documented germline pathogenic (C5) or likely pathogenic variant (C4) in either *BRCA1* or *BRCA2* genes was contacted by phone/email. The research purposes and the procedure were explained, and a request was made to share the research information with their first-degree male relatives and to invite them to participate in the research. If male relatives agreed to participate in the study, a researcher called them and shared an information sheet, recollected email address, and asked them to complete an informed consent form before starting the survey. The questionnaires have been implemented on QualtricsTM Platform via a link sent by email and available for two weeks. Other than being a male first-degree relative of a patient with *BRCA1/2* germline mutation, inclusion criteria were: (i) age 18+; (ii) no personal history of cancer; (iii) no previous *BRCA1/2* testing undergone. 

A pre–post test design was applied [36]. In the pre-test (T1) participants replied to questions on their socio-demographic data, health status, risk perceptions, outcome expectancies. Three weeks after completing the first questionnaire, a second link was shared via email evaluating intention to undergo *BRCA1/2* germline genetic screening and planning (T2). Participants’ data were pseudo-anonymized and data collection was performed through an ID code (i.e., a combination of letters and numbers). Overall recruitment lasted from September 2020 to July 2021. 

### 2.2. Participants 

The sample size has been determined through an a priori power analysis using GPower 4.0 [37]. Among the imputed parameters, it was chosen to include a prudential squared multiple correlations of 0.15 between each predictor and the outcome [38], alpha lower than 0.05, and power d (1-B) = 0.95. The final estimated number of participants is 103. 

The final sample of this study comprised 115 participants that were included in the analysis. All the participants were males, first-degree relatives of patients with an established germline genetic mutation (pathogenic or likely pathogenic variants) of the *BRCA1* and/or *BRCA2* genes, with ages ranging from 18 to 81 years old (M_age_ = 41.93; SD = 17.27). Regarding the education level, most of the respondents had a high school diploma (47%; N = 54), 33.9% had a bachelor or master’s degree (N = 39), and 19.1% had a middle school diploma (N = 22). Most of the participants were employed (78.3%; N = 90) and 47.8% (N = 55) of respondents had offspring, ranging from 1 to 6 children (M = 1.93; SD = 0.99). Regarding participants’ health condition, 30.4% were currently suffering from chronic illnesses (such as diabetes, hypertension, heart disease, or gastritis; N = 35). Finally, 38.3% and 23.5% of the respondents reported good (N = 44) or very good (N = 27) general health conditions, respectively.

Furthermore, the final sample of 115 participants was characterized by different degrees of kinship with the proband; specifically, the majority of participants have either a sister with *BRCA1/2* germline mutation (43.5%, N = 50) or the mother (41.7%, N = 48), while others had a daughter (7.8%, N = 9), or more than one relative with identified *BRCA1/2* variants (7%, N = 8). 

Regarding the proband’s cancer localization (N = 94), the majority were affected by breast cancer (83%; N = 78) or ovarian cancer (7.5%; N = 7), six probands were affected by both ovarian and breast cancer (6.5%), and only three were not affected but reported a significant family history of breast cancer (3%).

### 2.3. Measures

A self-administered questionnaire was applied to recollect information about different domains.

#### 2.3.1. T1

- Socio-demographic characteristics. Self-reported age, education, employment, parental status (0 = without children; 1 = with children), and degree of kinship with the proband with *BRCA1/2* germline mutation were assessed.
- Health Status. Self-reported general health and existing diagnosis for chronic disease were investigated with a single item each [39,40]. Response options for general health conditions were on a 5-point Likert scale, ranging from “very poor health conditions” to “very good health conditions”. Response option for chronic disease item was dichotomous (no–yes, specify).
- Risk perceptions. Relative risk perception regarding the possibility to develop breast, prostatic, and pancreatic cancer was investigated with one item each [41,42], on a 7-point Likert scale ranging from “far below the average” to “far above the average”. An example item is: “Compared to people like you by age and gender, your chances of having breast cancer in the future are…”.
- Outcome Expectancies. The outcome expectancies were assessed through the use of three different measures.
  (a) Self-referred Outcome Expectancies. Four items on a 5-point Likert scale from “unlikely” to “likely” were created ad hoc to evaluate participant self-referred positive outcome expectancies related to genetic testing (e.g., “The genetic test for *BRCA1/2* would increase my sense of safety about my health”). A total score had been calculated as a mean of the scores assigned to each item, with higher scores indicating higher self-referred outcome expectancies. The scale revealed a Cronbach’s alpha coefficient equal to 0.735 (rs > 0.488).
  (b) Family-referred Outcome Expectancies. Four items on a 5-point Likert scale from “unlikely” to “likely” were created ad hoc to evaluate participant family-referred positive outcome expectancies related to genetic testing (e.g., “My family members will have important information for their health if I undergo a *BRCA1/2* genetic test”). Total scoring is the mean of the points assigned to each item, with higher scores indicating higher family-referred outcome expectancies. The scale has been shown to be internally consistent (α = 0.862; rs > 0.689).
  (c) Perceived Benefit. Five-digit semantic differential questions asked where the respondent’s position was on a scale between two bipolar adjectives, with the aim to measure the perceived benefit for the genetic testing. As previously performed in other research [43], the scale was composed of 10 bipolar adjectives with higher scores indicating higher perceived genetic testing benefit. Examples of the proposed adjectives are “not important/important”, “irrelevant/relevant”, “useless/useful”, “unnecessary/necessary”. In this study, this scale has been shown to be internally consistent (α = 0.914, rs > 0.253).

#### 2.3.2. T2

- Intention. The intention to undergo genetic testing was measured through three items on a 5-point Likert scale from “very improbable” to “very probable”, evaluating the urge to engage the behavior [44]. Example of item is: “In the next 6 months, how likely is it that you will undergo *BRCA1/2* genetic testing?”. An overall score of intention had been calculated as a mean of the scores assigned to each item with higher scores indicating higher intention. The internal consistency of the scale was moderate (α = 0.694, rs > 0.408).
- Coping Self-efficacy. Three ad hoc items on a 5-point Likert scale, from “not very capable” to “totally capable”, were created to evaluate whether the individual feels to be capable of tackling the possible obstacles and difficulties that could make it difficult to undergo a genetic screening [45]. Total scoring is the mean of the points assigned to each item, with higher scores indicating higher coping self-efficacy. An example item is: “How do you feel you’re capable of tackling the obstacles and difficulties that could make it difficult for you to undergo genetic screening?”. In the present study, the scale revealed acceptable internal consistency (α = 0.773, rs > 0.525).
- Planning. Six ad hoc items were created to assess if participants planned the action to undergo *BRCA1/2* genetic testing (action planning—3 items), and if they developed one or more plans to cope with such a challenging situation that they think to encounter in planning the action (coping planning—3 items) [45]. Response options were on a 5-point Likert scale, ranging from “not true at all” to “very true” for the action planning dimension, and from “very improbable” to “very probable” for the coping planning dimension. An example item is: “I made plans about when to do a genetic screening for the *BRCA1/BRCA2* mutation (e.g., taking work permits)”. The total score had been calculated as a mean of the scores assigned to each item, with higher scores indicating higher planning. In this study, this scale has been shown to be internally consistent (α = 0.652; rs > 0.168).

### 2.4. Data Analysis Plan

Statistical analyses have been performed using the statistical software analysis package SPSS (Version 26.0, IBM, Armonk, NY, USA). Normality of the data was checked. Preliminary analyses included descriptive and Pearson’s r correlations between variables. An independent samples *t*-test was performed to further explore the differences in the levels of intention related to parental status. The psychological determinants of the intention were tested via hierarchical multiple regression analyses, as well as the predictive power of intention over planning. To investigate the impact of outcome expectancies on planning as mediated by the intention to undergo genetic testing, a simple mediation analysis was performed using PROCESS 3.4 macro for SPSS. The outcome variable for the analysis was the planning. The predictor variable for the analysis was the perceived benefit of the genetic testing. The mediator variable was the intention to undergo genetic testing. The covariate variables were age, parental status, and coping self-efficacy. Finally, one-way ANOVA with Bonferroni correction was carried out to further investigate results related to the risk perception of the breast cancer.

## 3. Results

### 3.1. Preliminary Results

Significant associations emerged between the intention to undergo *BRCA1/2* genetic testing and age, parental status, breast cancer risk perception, self-referred outcome expectancies, perceived benefit, coping self-efficacy, and planning. Table 1 shows means, standard deviations, and correlation coefficients between the variables. 

Intention showed a negative correlation with age and parental status. The *t*-test showed a significant difference between participants with and without children regarding their intention to uptake genetic testing (t (91) = 2.142, *p* = 0.035). Specifically, males without children showed higher intention (M = 3.51; SD = 0.836) compared to males with children (M = 3.13; SD = 0.879). Furthermore, intention showed a positive correlation with relative risk perception of breast cancer, self-referred outcome expectancies, and perceived benefit of genetic testing. No significant correlation was found between family-referred outcome expectancies and intention. Finally, intention showed a positive correlation with planning and coping self-efficacy. 

### 3.2. Regression Analysis

A simple linear regression was carried out to test whether the psychological variables that were significantly correlated with the outcome predicted the intention to undergo *BRCA1/2* genetic testing after three weeks, controlling for sociodemographic variables. Table 2 shows regression coefficients. The results of the regression indicated that the model was significant, F_(6,87)_ = 5.355, *p* < 0.001 and explained 28.4% of the variance. Specifically, it was found that the perceived benefit of genetic testing significantly predicted intention to uptake genetic testing (β = 0.30, S.E = 0.12, *t* = 2.71, *p* < 0.01). However, age, parental status, breast cancer risk perception, self-referred outcome expectancies, and coping self-efficacy were excluded from the model.

A linear regression analysis was run to verify whether intention to undergo *BRCA* testing and coping self-efficacy predicted the level of planning after three weeks, controlling for sociodemographic variables and other psychological variables. The results of the regression indicated that the model was significant, F_(2,87)_ = 3.03, *p* < 0.01 and explained 21% of the variance. Specifically, it was found that intention and coping self-efficacy predicted planning of the action (Intention: β = 0.26, S.E = 0.75, *t* = 2.22, *p* < 0.05; Coping self-efficacy: β = 0.31, S.E = 0.79, *t* = 2.91, *p* < 0.01).

### 3.3. Mediational Analysis

**Figure 1:**
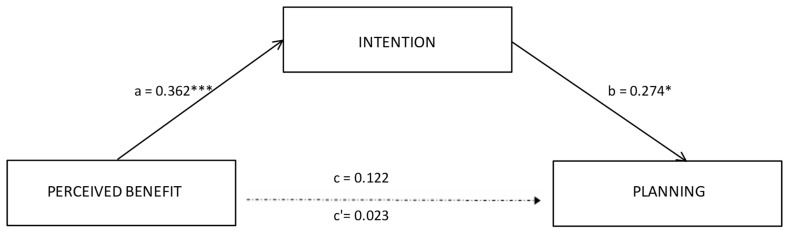
Results of the mediation model. Note: * *p* < 0.05; *** *p* <.001. Significant path coefficients are displayed as continuous lines; insignificant paths are displayed as dotted lines. The covariates are not displayed here, they were estimated in the model.

Based on the regression results, we tested mediation analysis with perceived benefit as the independent variable. The results showed that perceived benefit did not directly predict action planning (β = 0.02; SE = 0.07; *p* = 0.83). However, analyzing the indirect effect, the results revealed that intention significantly mediated the relationship between perceived benefit and planning, β = 0.10, SE = 0.06; 95% CI [0.009, 0.229]. Perceived benefits positively affected intention (β = 0.36, SE = 0.11; *p* < 0.001; 95% CI [0.184, 0.611]) and intention, in turn, positively affected action planning (β = 0.27, SE = 0.07; *p* < 0.01; 95% CI [0.029, 0.319]). Among the covariates, age predicts intention (β = 0.01, SE = 0.05; 95% CI [0.029, −0.002]. The results identified intention as a positive total mediator of the relationship between perceived benefit and planning. Considering the adjusted R^2^, this model explained 21% of the variance. Table 3 shows the direct, indirect, and total effect. Figure 1 showed the final mediation model, reporting significant path coefficients.

### 3.4. Breast Cancer Risk Perception

Considering that among relative risk perceptions, breast cancer is the only one that showed a significant positive correlation with the intention to undergo genetic testing, it was wondered what factors could influence the relative risk perception of breast cancer. Specifically, age showed a negative correlation with breast cancer risk perception (r = −0.216, *p* < 0.01), i.e., younger participants showed higher levels of perceived breast cancer risk.

Furthermore, there was a significant difference between groups based on the degree of kinship, as demonstrated by the one-way ANOVA (F _(3,111)_ = 3.68, *p* = 0.014). Bonferroni-corrected pairwise comparisons revealed that the group with more than one relative with *BRCA1/2* identified variant had a relative risk perception of breast cancer that was significantly higher (M = 4.38, SD = 0.51) than those who had a sister with an identified variant (M = 2.68; SD = 1.62). There was no statistically significant difference with the other groups (who has mother as a proband: M = 3.33; SD = 1.56; who has a daughter as a proband: M = 2.57; SD = 1.41). 

## 4. Discussion

To date, attention has been focused primarily on women in families with *BRCA1/2* mutations, due to the higher impact of those variants in terms of breast and ovarian cancer risk. The current study investigated the impact of psychological variables on the intention to uptake *BRCA1/2* genetic testing and its relationship with planning the action in men with a first-degree relative tested positive for that pathogenic variant. Principles from the HAPA theoretical model were applied.

Our hypotheses regarding the role of risk perceptions, outcome expectancies and coping self-efficacy in predicting the intention to uptake *BRCA1/2* genetic testing (HP1a) were partially supported. Specifically, the findings from the regression analysis evidenced that only the perceived benefit associated with the genetic testing may contribute to predict the intention to undergo *BRCA1/2* genetic testing. Therefore, rather than expectancies related to the self or the family, perceived benefit resulted as the key psychological determinant in influencing the intention to undergo *BRCA1/2* genetic testing for men.

Regarding HP1b, our results showed the role of the intention and coping self-efficacy in predicting the planning of the action, as supported by the HAPA model literature [26]. Furthermore, our research shows a relationship between perceived benefit and planning the action (undergo genetic testing) through the mediation of the intention in male with a first-degree relative tested that positive for *BRCA1/2* pathogenic variant. In particular, the greater the perceived benefits of undergoing a genetic test, the greater the intention and therefore the likelihood of planning this important screening behavior, adhering to cascade screening. This is another demonstration of the validity of the principles of the HAPA model applied to this research.

Moving on now to consider the association between intention and other factors, several interesting results should be discussed. Specifically, higher breast cancer risk perception as well as higher perceived benefit and coping self-efficacy were found to be directly associated with higher intention. Moreover, beliefs that *BRCA1/2* genetic testing has more positive consequences and outcomes for one’s personal life was associated with higher intention, showing that the higher the self-referred anticipated consequences of engaging in *BRCA* genetic testing, the higher the intention experienced (RQ1). However, regarding RQ1 and in contrast to earlier qualitative findings [23,25,46] that supported the notion that men uptake *BRCA1/2* testing to protect their family (specifically the offspring) rather than to ascertain the risks to themselves, no evidence of association between family-referred outcome expectancies and intention to uptake *BRCA* genetic testing was detected. Related to this result and contrary to the expectations, in our study, males without children showed significantly higher intention than that shown by males with children. This latter result might be also connected to the relationship between age and intention. Indeed, in our study, younger participants, who are less likely to have offspring, showed higher levels of intention to adhere to cascade screening. Notwithstanding, future studies should investigate and verify these results by comparing males already tested and not yet tested. The participants’ recruitment of the previous studies was consequent to their *BRCA1/2* genetic testing and therefore also after genetic counseling sessions, where genetic counselors had the aim, among others, to help people in understanding and adapting to familial implications of genetic information. Our hypothesis is that in a pre-intentional phase, such as the one analyzed in our study, in which the subjects have not benefited from any genetic counseling session, males may be more focused on self-referred implication without the initial awareness of the family ones.

Additionally, age was found to be negatively associated with the intention to undergo *BRCA1/2* genetic testing, which was higher in younger males than older ones. Therefore, young age acted as a facilitating factor for the intention to undergo genetic testing. The result of this study is consistent with the literature, which identifies younger people with higher interest and more positive attitudes in genetic testing [29,47,48,49]. This result may also depend on the greater awareness of younger participants who have been exposed to professional communication about risk for years and, maybe, have higher knowledge.

Regarding risk perception and consistent with RQ2, beliefs that personal risk perception of breast cancer is higher than other people similar for age and gender were associated with higher intention to undergo genetic screening for *BRCA1/2*. Some studies seem to suggest the presence of an increased perception of cancer risk in healthy men tested for *BRCA1/2* (i.e., with no previous cancer diagnosis), irrespective of the genetic test result [50]. Interestingly, among the risk perceptions, in our study, breast cancer is the only one associated with the intention to undergo the test. Feeling at risk of breast cancer for a man could be emotionally activating, as a result of the gendered construction of breast cancer as a women’s disease [20], impacting on the intention to undergo preventive screening behaviors. Furthermore, when there is a symptomatic family history of multiple breast cancer in first degree-relatives, men should express concern about developing breast cancer themselves [20,50]. Indeed, data concerning cancer localization in the probands showed that almost all of them were affected by breast cancer. This may have influenced our male participants’ perception of risk. Furthermore, the higher perceived risk for breast cancer may be related to the fact that participants have not yet undergone genetic counseling and therefore may not be well informed about the higher risk in males for prostate cancer and others, such as pancreatic cancer and/or melanoma.

Interestingly, excluding participants who have more than one relative with identified *BRCA* mutation, who perceived their cancer risk as average, the others (with different degrees of kinship with the proband) perceived themself as less at risk than the average population of equal gender and age. Studies support the notion that people who are in a state of higher risk such as that determined by an identified genetic mutation perceive themselves to be less at risk of cancer than the rest of the population, that is, to reduce the distress that this awareness would cause them, applying a cognitive regulation strategies of distress reduction [51]. Additionally, first- and second-degree relatives of probands with hereditary cancer syndrome perceived their relative cancer risk as being lower than their peers of the same age and gender [52,53]. If risk perceptions appeared as potential emotion regulation strategies used by individuals exposed to an increased risk, it remains an open question whether these could have an implication for compliance and adherence with possible cancer surveillance programs [54,55]. Future studies may investigate the link between cancer risk perception and surveillance compliance in this target population of high-risk males.

Furthermore, in our sample, a negative association was found between age and perceived breast cancer risk, highlighting that younger men reported higher levels of breast cancer risk perception. In our sample, the youngest participants were generally children of probands. As supported by Liede et al. [50], a higher risk perception of cancer (breast cancer included) was strongly associated with a diagnosis of breast or ovarian cancer in the mother or with the mother’s death in men tested for *BRCA* mutations. Cancer risk perception is influenced by the experience of cancer disease within the family [56,57,58], as well as by cancer patient’s experience (side effects of chemo/hormonal therapy, physical deterioration, depression, anxiety, use of invasive procedures). Furthermore, data concerning male participants’ role in the management of the patient’s disease (e.g., caregiving) were not collected; however, it would be useful data to gather in future studies because it may also be involved in the formulation of risk perception. These considerations supported the idea that both cognitive and emotional factors interplay in the formulation of cancer risk perception [59].

Despite the methodology applied in the present research and the pre–post test design, some limitations should be noted. First, this study included a small number of participants that are vulnerable to self-selection bias, particularly because the available probands were involved in contacting their male first-degree relatives. It might be that the probands who agreed to participate were the most willing to share health information with their male relatives and the most willing to involve them in the mutation identification process, as were the men who decided to participate in the study. Second, although based on HAPA model literature, there is a proven strong link between intention and planning, and between planning and subsequent implementation of the target behavior, data on the action of undergoing genetic testing itself were not collected. Future studies should test how the psychological factors identified as salient for intention affect the actual enactment of the genetic screening in a cascade screening context. Third, the present research did not test the level of the knowledge, or subjective and objective health literacy [60] of our participants related to the *BRCA1/2* genetic testing or any other exposure to information that the participants could have searched for themselves. The measures applied in the present research had the aim to collect information regarding possible psychological variables influencing intention to adhere to prescribed guidelines for *BRCA1/2* genetic testing not to inform their decision-making. Participants were not exposed to any kind of genetic counseling session before entering the study. They might not be fully aware about all the implications connected to the *BRCA1/2* germline mutations, and this may have influenced their intention to adhere to the guidelines for relatives of *BRCA1/2* proband. Fourth, the measure of expectation of the outcome of the genetic test (i.e., self-referred and family-referred outcome expectation) was worded in a positive way only and did not consider the possible difference between short- and long-term beneficial vs. detrimental expectations. Future research should investigate these points with a more sensible measure.

## 5. Conclusions

The study has some peculiar strengths. Specifically, in contrast to previous studies involving first- to third-degree relatives, our study focused on a homogeneous sample of first-degree relatives only, who therefore share the same exposure to the risk (50%) of having inherited or passed on that pathogenic variant. Furthermore, our sample consists of subjects who have not previously undergone genetic counseling sessions. Therefore, their knowledge and attitudes have not yet been influenced by any formal educational intervention that provides the basis for informed decision [61] regarding *BRCA1/2* genetic testing. On a theoretical level, our findings partially supported the Health Action Process Approach as a valuable model based on which to develop interventions in the context of cascade screening for *BRCA1/2* genetic testing. Specifically, the most important factor influencing the intention to adhere to the genetic testing guidelines for relatives of *BRCA1/2* proband seems to be the perceived benefit of testing, while planning of the action results from the intention itself and the coping self-efficacy. Those results seem to stress the importance of integrated genetic counselling sessions with a strict collaboration between genetics and psychologists together with interventions planned to increase men’s self-monitoring ability to support their self-efficacy.

## Figures and Tables

**Table 1:** Means, standard deviations, and correlations between the main variables in the study.

Variables	M (SD)	1	2	3	4	5	6	7	8	9	10	11
1. Age	41.93 (17.28)	−	0.717 **	−0.220 *	0.216 *	−0.043	0.002	0.231 *	0.171	−0.237 *	−0.006	−0.082
2. Parental Status ^	-		-	−0.235 *	0.137	0.034	0.074	0.246 **	0.144	−0.223 *	−0.039	−0.076
3. Breast Cancer Risk Perception	3.07 (1.59)			-	0.413 **	0.519 **	−0.044	−0.070	0.020	0.227 *	0.149	0.129
4. Prostate Cancer Risk Perception	3.88 (1.11)				-	0.680 **	0.155	0.164	0.095	−0.072	−0.024	0.003
5. Pancreatic Cancer Risk Perception	3.52 (1.20)					-	0.138	0.056	0.090	0.037	−.016	0.189
6. Self-referred Outcome Expectancies	4.11 (0.72)						-	0.573 **	0.521 **	0.237 *	0.104	0.065
7. Family-referred Outcome Expectancies	4.17 (0.84)							-	0.377 **	0.052	0.126	−0.003
8. Perceived Benefit	4.05 (0.84)								-	0.320 **	0.173	0.199
9. Intention	3.34 (0.87)									-	0.370 **	0.272 **
10. Planning	3.07 (0.57)										-	0.406 **
11. Coping Self-Efficacy	3.56 (0.74)											-

* *p* < 0.05; ** *p* < 0.01. Correlation coefficients are Pearson’s r except for ^ Spearman’s Rho.

**Table 2:** Regression tables.

	Dependent Variables					
	Intention				Planning	
	β	*p*			β	*p*
Step 1: Sociodemographic						
Age	−0.231	0.102			−0.009	0.949
Parental Status	−0.102	0.468			−0.041	0.789
$F_{\left(2,87\right)}\;$= 4.51 *; R^2^ = 9.6%			$F_{\left(2,87\right)}\;$= 0.09; R^2^ = 0.2%			
Step 2: Psychological variables						
Age	−0.243	0.071			0.022	0.881
Parental Status	−0.088	0.500			−0.025	0.860
Breast Cancer Risk Perception	0.127	0.205			0.103	0.342
Self-referred Outcome Expectancies	0.116	0.297			0.059	0.620
Perceived Benefit	0.305	0.008 **			0.094	0.446
Coping Self-Efficacy	0.126	0.198			0.334	0.002 **
$F_{\left(6,87\right)}\;$= 5.35 ***; R^2^ = 28.4%			$F_{\left(2,87\right)}\;$= 2.585 *; R^2^ = 16.1%			
Step 3 Intention						
Age	-	-			0.085	0.555
Parental Status	-	-			−0.002	0.989
Breast Cancer Risk Perception	-	-			0.070	0.513
Self-referred Outcome Expectancies	-	-			0.029	0.804
Perceived Benefit	-	-			0.014	0.911
Coping Self-Efficacy	-	-			0.301	0.005 **
Intention	-	-			0.261	0.029 *
			$F_{\left(2,87\right)}\;$= 3.03 **; R^2^ = 21%			

* *p* < 0.05; ** *p* < 0.01; *** *p* < 0.001.

**Table 3:** Mediation analyses.

Independent Variable: Perceived Benefit Mediator: Intention			
Dependent Variable	Direct Effect	Indirect Effect	Total Effect
Planning	β = 0.02	β = 0.10	β = 0.12
	SE = 0.07	SE = 0.06	SE = 0.07
	[95% CI] = −0.137, 0.169	[95% CI] = 0.009, 0.229	[95% CI] = −0.061, 0.231

## Data Availability

The data presented in this study are available on request from the corresponding author. The data are not publicly available.
